# Self-presentation in Online Professional Networks: Men's Higher and Women's Lower Facial Prominence in Self-created Profile Images

**DOI:** 10.3389/fpsyg.2017.02295

**Published:** 2018-01-17

**Authors:** Sabine Sczesny, Michèle C. Kaufmann

**Affiliations:** Department of Psychology, University of Bern, Bern, Switzerland

**Keywords:** face-ism, body-ism, gender, appearance self-esteem, social media, self-presentation

## Abstract

Men are presented with higher facial prominence than women in the media, a phenomenon that is called *face-ism*. In naturalistic settings, face-ism effects could be driven by gender biases of photographers and/or by gender differences in self-presentation. The present research is the first to investigate whether women and men themselves create this different facial prominence. In a controlled laboratory study, 61 participants prepared a picture of themselves from a half-body photograph, allegedly to be uploaded to their profile for an online professional network. As expected, men cropped their photos with higher facial prominence than women did. However, women and men did not differ in the self-presentational motivations, goals, strategies, and personality variables under investigation, so that the observed face-ism effect could not be explained with these variables. Generally, the higher participants' physical appearance self-esteem, the higher was their self-created facial prominence.

## Introduction

Gender differences exist in how the face is presented in relation to the body in pictures in the media: Men's heads and faces are presented in greater detail in media portrayals than they are for women; this phenomenon was termed “face-ism” (Archer et al., 1983). In research that focuses on the (sexual) objectification of women in the media (e.g., in advertisement) the phenomenon was termed “body-ism,” documenting the greater focus on women's bodies or body parts (Unger and Crawford, 1992). The present research investigates the relative face-to-body ratio in women's and men's pictures in online professional networks, a context in which photographs usually range from half-figure pictures to portraits. Most importantly, first impressions in person perception (Zebrowitz, 2006; Freeman and Ambady, 2011), such as those evoked by pictures in social media, influence crucial real-world outcomes such as choices of whom to date, befriend, or employ (White et al., 2017). Since higher facial prominence is associated with higher ascriptions of intelligence, competence, dominance, mental activity, and morality (Archer et al., 1983; Schwarz and Kurz, 1989; Zuckerman and Kieffer, 1994; Loughnan et al., 2010), it is important to pay special attention to the pictures that are used for self-presentation.

In their early media analyses, Archer et al. (1983) examined pictures of women and men in American newspapers, in works of art and photographs from several countries as well as in amateur drawings. Their investigation confirmed that men were more likely to be shown with a focus on the face, women with an emphasis on the body. This gender difference was consistently found in the mass media, namely in newspapers, magazines, television, and the Internet (e.g., Sparks and Fehlner, 1986; Nigro et al., 1988; Copeland, 1989; Dodd et al., 1989; Iijima Hall and Crum, 1994; Konrath and Schwarz, 2007; the effect may be restricted to certain magazines: Cheek, 2016). With regard to social media, past research documented face-ism effects on social networking platforms such as Facebook, MySpace, VKontakte, etc. (Reichart Smith and Cooley, 2012; Cooley and Reichart Smith, 2013) and on official websites of politicians and professors (Konrath and Schwarz, 2007; Szillis and Stahlberg, 2007; Konrath et al., 2012).

Archer et al. (1983) assumed that this gender asymmetry in facial prominence “may lie deep in historic conceptions about essential differences between the sexes” (p. 734), in that men are associated with mental qualities and women with somatic qualities such as physical attractiveness. Other researchers proposed that social roles of the depicted person function as a moderator of facial prominence (e.g., Sparks and Fehlner, 1986; Dodd et al., 1989), supported by the idea that face-ism may vary with (occupational) status (e.g., Matthews, 2007). Age, which is also often linked with status, was found to influence facial prominence as well. For example, faculty members displayed their faces more prominently than students (Read et al., 2017), and older women displayed their faces more prominently than younger women (Szillis and Stahlberg, 2007; Read et al., 2017). However, the underlying psychological mechanisms of face-ism in self-presentation are not yet fully understood.

In the existing naturalistic studies in social media contexts, face-ism effects might have been driven by gender biases of photographers and/or created by the depicted individuals themselves. People generally attempt to influence the impression they make on others and to ensure that their public image suits the demands of a particular situation (i.e., impression management; Leary and Kowalski, 1990; Leary, 1995). Having full control over their self-presentation in social media, people may highlight desirable aspects of themselves and conceal unwanted ones (Mendelson and Papacharissi, 2010). This may also include determining facial prominence in media profiles by choosing and editing their images.

The first aim of the present research was therefore to determine—under improved experimentally controlled conditions (and hence accepting a decreased ecological validity)—whether men and women themselves create face-ism in their self-presentation for an online professional network. We expected men to create higher and women lesser facial prominence in their profile pictures for the network (Hypothesis 1).

The second aim of the research was to examine whether differences in women's and men's impression management help to explain face-ism (body-ism) effects. The impression management of women and men may be influenced by their gender identities: women view themselves as more communal, whereas men view themselves as more agentic (see meta-analysis by Donnelly and Twenge, 2017). Communion refers to the maintenance of social relationships (e.g., benevolence, trustworthiness), while agency refers to goal-achievement (e.g., competence, assertiveness; Abele and Wojciszke, 2014). Past research has shown that various gender differences in self-presentation are in line with gender identities, more recently also with respect to social networking sites:

Men tend to engage in self-enhancement/self-promotion more often than women by emphasizing their best characteristics (Guadagno and Cialdini, 2007), they are also more likely to use assertive self-presentational strategies than women (Lee et al., 1999). Moreover, the fact that men are more narcissistic than women indicates their higher need for admiration and power (Grijalva et al., 2015). In a nationally representative sample of U.S. men, narcissists reported editing photos of themselves more frequently (i.e., cropping or cutting parts of themselves out of pictures, using photographic filters, and using picture editing software and applications) for photos they posted to social networking sites (i.e., on Facebook, Twitter, Instagram, Tumblr, and Pinterest; Fox and Rooney, 2015). Furthermore, men are less concerned about their physical appearance (e.g., Dion et al., 1990; Gillespie and Eisler, 1992) and report higher appearance self-esteem (e.g., Gentile et al., 2009) than women. Men's higher acceptance of and confidence in their physical appearance may enable them to present their faces prominently, even given blemishes or defective features, whereas women's lower acceptance of and confidence may result in deflecting perceivers' attention away from potential facial imperfectness, which reduces facial prominence.

These gender differences in motivations, self-presentational strategies, and/or related personality attributes may help to understand why men present themselves with more detailed views of their faces than women. In doing so, both genders can ensure that their public image suits their gender identities and confirm gender-stereotypic expectations (Prentice and Carranza, 2002). Hence, we expected gender differences in facial prominence to be due to men's higher motivation and use of distinct strategies to be perceived as competent, assertive, and intelligent, their higher need for admiration and power (narcissism) and their higher appearance self-esteem (Hypothesis 2). Furthermore, we explored what participants intended to achieve with their photograph in the professional network (free response question regarding their goals).

To sum up, gender differences in face-to-body ratios in media representations appear to be a pervasive phenomenon. This may have important implications for the individuals concerned, as higher facial prominence is associated with higher ascriptions of intelligence, competence, dominance, mental activity and morality. To date, it remains an open question whether decisions and actions of the depicted individuals themselves contribute to face-ism in social media contexts, and if so, which psychological mechanisms underlie the phenomenon in the context of self-presentation in online professional networks.

## Methods

### Participants

The sample consisted of 61 university students (32 women and 29 men) studying various subjects. Most participants (98%) attended the University of Bern, Switzerland. They were recruited in cafeterias and libraries. Their mean age was 24.08 years (ranging from 19 to 33 years).

To determine the sample size, we used G^*^Power 3, a statistical power analysis program (Faul et al., 2007). The power analysis (high effect size of *d* = 0.75; α-level 0.05; power of 0.8; 2 groups) indicated a sample size of 58 participants.

### Materials

The participants received all instructions and materials on a computer in the laboratory. In the introduction of the study, we asked participants to take part in a study on “how to present oneself in the best possible way with a picture on a professional network” (cover story). We then provided them with a screenshot of the starting page of a fictitious professional network. The webpage was developed for this study and looked like a typical registration website of existing professional networks, including input boxes to provide personal data (e.g., name, email address, password). Next, we provided them with a screenshot of an empty member profile of the network, including input boxes to provide more personal information (e.g., education, training). This profile also contained a space for inserting the picture. Afterwards, we took two half-body photographs of the participants and asked them to choose the photograph they believed represented them best. We then presented the photograph they had chosen on the screen. The instruction reads as follows: “We now ask you to crop the photograph that you selected for your profile. Please use the program which has been opened to prepare the picture that represents you best and then save the picture.”

**Figure 1:**
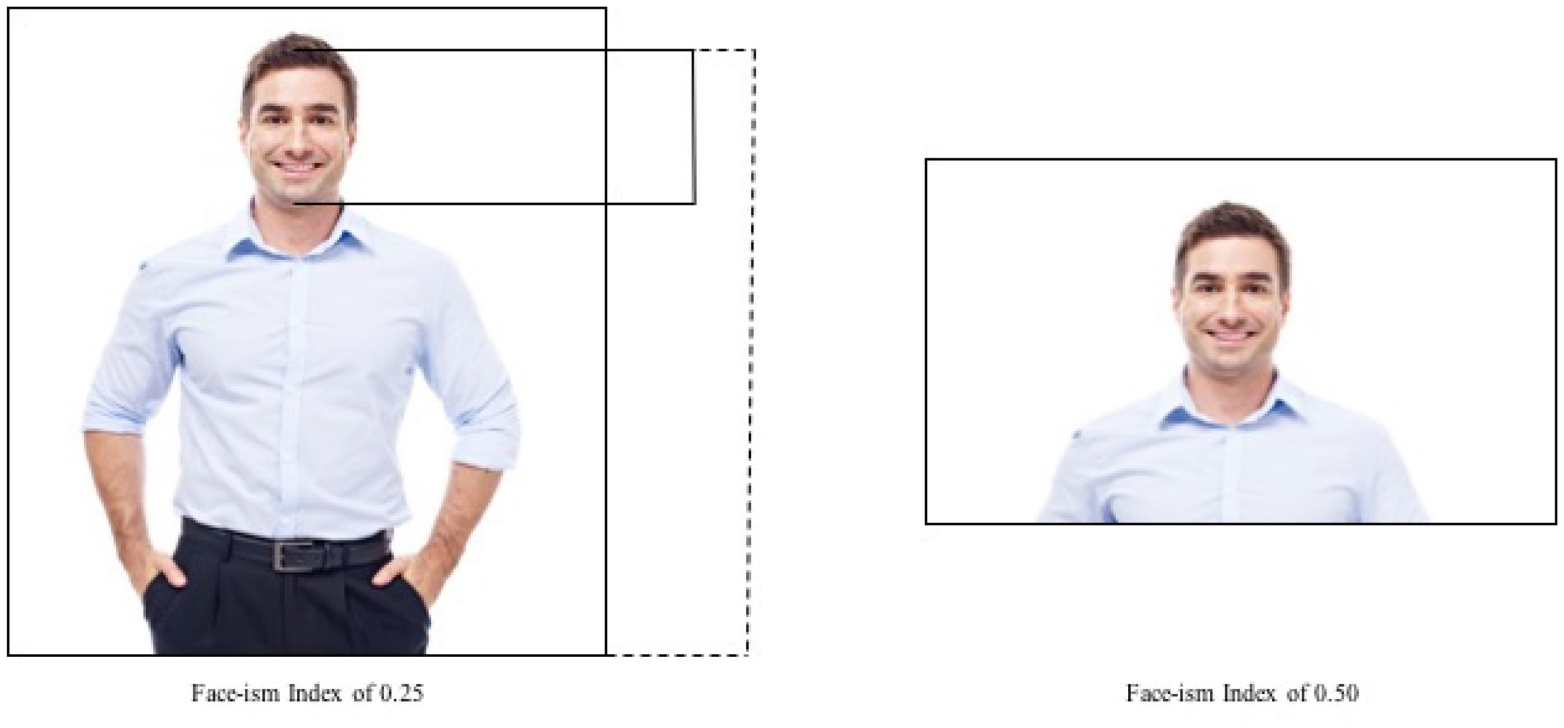
Illustrates two different face-ism indices. Illustration of face-ism indices (using an iStock photograph). Solid line is the numerator, dotted line the denominator. Face-ism = Numerator/Denominator.

Two independent researchers measured the prominence of the face in all pictures on printouts, independently of each other. The size of all pictures was 3,872 × 2,592 pixels with a resolution of 300 pixels. Both researchers used the same ruler and calculated *face-ism indices* for all pictures (Archer et al., 1983). The index was based on the ratio of two linear measurements (see Figure 1): (1) “the numerator,” i.e., the distance from the top of the head to the end of the chin, and (2) “the denominator,” i.e., the distance from the top of the head to the lowest visible part of the subject's body. The resulting values vary from 0 (only the body is depicted) to 1 (only the face/head is visible in the picture). Consequently, the higher the index, the more space is devoted to the face. A comparison of the measurements of both researchers revealed almost identical results for all pictures. The overall interrater reliability Fleiss Kappa was κ = 0.98.

After they had cropped their pictures, we asked the participants about their goals. They described in their own words what they intended to achieve in the professional network with the help of their photograph (free response question). Two independent raters classified the answers. Independent of each other, the two raters set up the following nine categories, namely (1) make a competent impression (e.g., “exude competence,” “make a professional appearance”), (2) give a first impression (e.g., “give a first impression,” “show others what I look like”), (3) make a likable impression (e.g., “appear likable,” “leave a friendly impression”), (4) attract attention (e.g., “gain attention,” “arouse interest”), (5) be identifiable (e.g., “show what I look like,” “recognition”), (6) make an attractive/well-groomed impression (e.g., “appear attractive,” “well-groomed appearance”), (7) make an authentic/natural impression (e.g., “appear authentic”, “leave a natural impression”), (8) meet context demands (e.g., “presenting myself is part of my CV,” “provide an impression that suits the network”), and (9) make a trustworthy impression (e.g., “trustworthy appearance,” “inspire trust”). The raters assigned each goal to one of these categories. The overall interrater reliability Fleiss Kappa was κ = 0.75. In the few cases where their decisions diverged, they discussed the goal and agreed on a joint categorization.

Participants also answered 18 questions (on 7-point scales) that measured their impression management motivation, assertive self-presentation strategies, narcissism, as well as appearance self-esteem, presented in random order:

*Impression management motives* were measured with six self-developed items: “How much do you wish to highlight your competence/assertiveness/intelligence, attractiveness/the attractiveness of your face/the attractiveness of your body in this photograph?” (*not at all, very much*).

*Assertive self-presentation strategies* were measured with six items (taken from the *self-presentation tactic scale*; Lee et al., 1999): “When I succeed at a task, I emphasize to others how important the task was” (*Enhancement*), “I try to set an example for others to follow” (*Exemplification*), “I point out the positive things I do which other people fail to notice” (*Entitlement*), “I use my size and strength to influence people when I need to” (*Intimidation*), “I express opinions that other people will like” (*Ingratiation*), and “I exaggerate the negative qualities of people who compete with me” (*Blasting*) (*disagree, agree*).

*Narcissism* was measured with three items (taken from of the *Narcissistic Personality Inventory*, Raskin and Terry, 1988; German translation by Schütz et al., 2004): “I like to be the center of attention,” “I find it easy to manipulate people,” and “I like to have authority over other people” (*disagree, agree*; Cronbach's α = 0.71).

*Appearance self-esteem* was measured with three items (taken from Dion et al., 1990): “I am self-conscious about the way I look,” “I am unconcerned with how others feel about my appearance,” and “I worry about how others are evaluating how I look (reversed)” (*disagree, agree*; Cronbach's α = 0.57).

Finally, since men were found to evaluate their photographs as more attractive than women (Costa and Bitti, 2000), we asked participants to evaluate their *satisfaction with the picture* they had prepared from one of the two original half-body photographs: “Please answer some questions concerning the photograph that you edited. In your opinion, how well does that picture represent you?” (*not well, very well*), “How satisfied are you generally with your picture?” (*not satisfied, very satisfied*), “How satisfied are you with the clothes you are wearing in the picture?” (*not satisfied, very satisfied*), “How attractive do you consider yourself in the picture?” (*not attractive, very attractive*), “Do you consider the picture suitable for the network?” (*not suitable, very suitable*), and “How likely would you use this photograph for the network?” (*not likely, very likely*). The responses were combined into a *satisfaction with picture* scale (Cronbach's α = 0.79).

### Procedure

Participants were recruited on campus and were accompanied to the lab by one male experimenter. Each participant gave informed written consent prior to being tested. The ethical committee of the University of Bern approved the study as being risk-free for participants and as maintaining their anonymity. In the online questionnaire we asked participants for personal data (i.e., sex, age, nationality, field of study, and/or profession). Then, another male experimenter took their photographs while they were standing on a marked line in front of a white wall. They were instructed not to move, to look at the lens, to keep their facial expression neutral, and to place their hands at the side of their bodies. Afterwards, participants were seated in front of a computer again. They were instructed to prepare the picture and to indicate their satisfaction with the picture. Then they continued to answer the questionnaire regarding their motivations, strategies, and personality attributes. Finally, participants were thanked, received a beverage and a chocolate bar for their participation and were debriefed. None of the participants was able to guess the hypothesis of the study.

## Result

Throughout this study, *p*-values of 0.05 or less were considered significant. Means, standard deviations, and correlations of all variables are presented in Table 1.

**Table 1:** Means, standard deviations, and correlations of face-ism, satisfaction with picture, self-presentational motivations, assertive self-presentational strategies, appearance self-esteem and narcissism.

	**Variables**	**N**	**M**	**SD**	**1**	**2**	**3**	**4**	**5**	**6**	**7**	**8**	**9**	**10**	**11**	**12**	**13**	**14**	**15**	**16**
1	Face-ism	61	0.54	0.13	–	−0.00	0.01	−0.08	0.18	0.09	0.2	−0.02	0.04	0.1	−0.24	−0.11	−0.04	0.01	0.28^*^	0.07
2	Satisfaction with Picture	61	3.09	0.94		–	0.16	0.07	0.08	0.13	−0.01	0.23	0.09	0.04	−0.08	0.11	0.04	−0.11	0.41^**^	−0.05
3	Motivation: Facial Attractiveness	61	4.56	1.56			–	0.37^**^	0.38^**^	0.22	0.34^**^	0.65^**^	−0.02	−0.02	−0.03	−0.06	0.50^**^	0.06	0.11	0.06
4	Motivation: Bodily Attractiveness	61	2.48	1.22				–	0.21	0.15	0.28^*^	0.43^**^	0.28^*^	0.25	0.2	−0.05	0.26^*^	0.14	−0.04	0.30^*^
5	Motivation: Assertiveness	61	3.31	1.56					–	0.59^**^	0.73^**^	0.51^**^	−0.04	0.00	0.07	0.23	0.08	−0.09	0.13	0.10
6	Motivation: Competence	61	4.20	1.70						–	0.77^**^	0.46^**^	−0.16	−0.24	0.11	0.21	0.13	−0.11	0.05	−0.10
7	Motivation: Intelligence	61	3.80	1.72							–	0.49^**^	−0.13	−0.05	0.09	0.21	0.04	0.03	0.06	0.02
8	Motivation: Attractiveness	61	3.51	1.56								–	0.08	0.12	0.02	0.14	0.34^**^	0.18	0.00	0.06
9	Strategy: Ingratiation	61	3.92	1.57									–	0.32^*^	0.2	0.04	−0.03	0.32^*^	−0.17	0.30^*^
10	Strategy: Intimidation	61	3.20	1.85										–	0.24	0.05	−0.07	0.22	−0.02	0.50^**^
11	Strategy: Entitlement	61	4.20	1.42											–	0.25	0.07	0.22	−0.42^**^	0.34^**^
12	Strategy: Enhancement	61	3.21	1.39												–	−0.18	0.03	−0.19	−0.06
13	Strategy: Exemplification	61	4.85	1.39													–	−0.08	0.03	0.17
14	Strategy: Blasting	61	2.59	1.54														–	−0.26^*^	0.28^*^
15	Appearance Self-Esteem	61	4.09	1.15															–	−0.08
16	Narcissism	61	3.44	1.32																–

Face-ism indices vary from 0.25 to 1. Higher values represent higher scores on the variables.

* p < 0.05;

** *p < 0.01*.

First, to rule out that the experimenter created face-ism when taking the original half-body photographs, we calculated face-ism indices for the original photographs that the participants had chosen as the basis for their picture. The values for the original photographs varied from 0.23 to 0.33 (for women: *M* = 0.28, *SD* = 0.02; for men: *M* = 0.27, *SD* = 0.02; *t*_(59)_ = 1.99, *p* = 0.051; *d* = 0.51) and indicated a tendency of higher facial prominence for female participants than for male participants, thus working in the opposite direction of the proposed face-ism effect.

Second, to rule out gender differences in participants' satisfaction with their self-created picture, we conducted an independent *t*-test. We found that women (*M* = 3.08, *SD* = 1.03) and men (*M* = 3.10, *SD* = 0.84) were similarly satisfied with their photographs, *t*_(59)_ = −0.08, *p* = 0.936, *d* = 0.02.

In line with Hypothesis 1, male participants (*M* = 0.58, *SD* = 0.14) created pictures with a higher facial prominence than women (*M* = 0.51, *SD* = 0.12), *t*_(59)_ = −2.08, *p* = 0.042, *d* = 0.54.

To test Hypothesis 2, we compared women's and men's impression management motivations, assertive self-presentational strategies, narcissism, and appearance self-esteem. In contrast to past research, women and men did not differ significantly in the variables under investigation (see Table 2). Men tended to present themselves as more intimidating than women (i.e., using their size and strength to influence people). The exploration of participants' self-presentational goals (free response question) revealed that most of them used the picture to achieve a competent impression, provide a first impression of themselves, to appear likable and/or attract attention; women tended to aim for a likable impression more often than men (see Table 3). In sum, in the present sample differences in women's and men's face-ism index cannot be explained by gender differences in the variables under investigation. Only appearance self-esteem correlated significantly with participants' face-ism index: The higher participants' physical appearance self-esteem, the higher was their self-created facial prominence (see Table 1). When calculated separately for women and men, however, the correlations were no longer significant (women: *r* = 0.26; men: *r* = 0.27).

**Table 2:** Comparing women and men: means, standard deviations, and *t*-tests for self-presentational motivations, assertive self-presentational strategies, appearance self-esteem and narcissism.

**Variables**	**M women**	**M men**	***t*-tests**
Motivation: Facial Attractiveness	4.66	4.45	*t*_(59)_ = 0.52, *p* = 0.606
	(1.64)	(1.48)	
Motivation: Bodily Attractiveness	2.38	2.59	*t*_(59)_ = −0.67, *p* = 0.504
	(1.24)	(1.21)	
Motivation: Assertiveness	3.25	3.38	*t*_(59)_ = −0.32, *p* = 0.749
	(1.50)	(1.64)	
Motivation: Competence	4.41	3.97	*t*_(59)_ = 1.01, *p* = 0.316
	(1.70)	(1.70)	
Motivation: Intelligence	3.81	3.79	*t*_(59)_ = 0.04, *p* = 0.965
	(1.77)	(1.70)	
Motivation: Attractiveness	3.69	3.31	*t*_(59)_ = 0.95, *p* = 0.349
	(1.55)	(1.56)	
Self-Presentation Strategy: Ingratiation	3.72	4.14	*t*_(59)_ = −1.04, *p* = 0.303
	(1.51)	(1.64)	
Self-Presentation Strategy: Intimidation	2.81	3.62	*t*_(59)_ = −1.73, *p* = 0.089
	(1.55)	(2.08)	
Self-Presentation Strategy: Entitlement	4.16	4.24	*t*_(59)_ = −0.23, *p* = 0.818
	(1.14)	(1.70)	
Self-Presentation Strategy: Enhancement	3.47	2.93	*t*_(59)_ = 1.52, *p* = 0.133
	(1.48)	(1.25)	
Self-Presentation Strategy: Exemplification	5.03	4.66	*t*_(59)_ = 1.06, *p* = 0.295
	(1.28)	(1.50)	
Self-Presentation Strategy: Blasting	2.38	2.83	*t*_(59)_ = −1.15, *p* = 0.256
	(1.54)	(1.54)	
Appearance Self-Esteem	3.97	4.22	*t*_(59)_ = −0.85, *p* = 0.401
	(1.19)	(1.11)	
Narcissism	3.18	3.72	*t*_(59)_ = −1.64, *p* = 0.107
	(1.23)	(1.38)	

*N = 61 with 32 women and 29 men. Standard deviations in parentheses*.

**Table 3:** Frequencies, percentages and chi square-tests for women's and men's goals they want to achieve in the professional network with their picture (answers to the free response question; presented in descending order).

**Goals**	***N***	**Percentage**	**Women**	**Men**	**Chi square-tests (two-sided)**
Competent impression	24	39.3%	13	11	$X_{\left(1\right)}^{2}$ = 0.046, *p* = 0.830
First impression	22	36.1%	10	12	$X_{\left(1\right)}^{2}$ = 0.677, *p* = 0.411
likable impression	17	27.9%	12	5	$X_{\left(1\right)}^{2}$ = 3.106, *p* = 0.078
Attracting attention	12	19.7%	5	7	$X_{\left(1\right)}^{2}$ = 0.698, *p* = 0.404
Being identifiable	8	13.1%	5	3	$X_{\left(1\right)}^{2}$ = 0.372, *p* = 0.542
Attractive/well-groomed impression	8	13.1%	5	3	$X_{\left(1\right)}^{2}$ = 0.372, *p* = 0.542
Authentic/natural impression	7	11.5%	4	3	$X_{\left(1\right)}^{2}$ = 0.070, *p* = 0.792
Suitable to context	5	8.2%	3	2	$X_{\left(1\right)}^{2}$ = 0.124, *p* = 0.725
Trustworthy impression	4	6.6%	2	2	$X_{\left(1\right)}^{2}$ = 0.010, *p* = 0.919

*N = 61 with 32 women and 29 men*.

## Discussion

This research represents the first attempt to determine whether women and men themselves create face-ism in their profile pictures for a professional network in social media. The strengths of this research are that participants themselves created their images in a controlled laboratory setting, which allowed for determining causality, and that gender differences in self-presentational motivations, goals and strategies, appearance self-esteem, and narcissism were taken into consideration, in order to examine underlying psychological mechanisms of face-ism effects.

The results of the laboratory study document that men indeed create more and women less facial prominence in their profile pictures for a professional network (see Hypothesis 1). But in contrast to past research and our assumptions, we did not find any significant gender differences in participants' impression management motivations, assertive self-presentation strategies, narcissism, and appearance self-esteem (see Dion et al., 1990; Gillespie and Eisler, 1992; Lee et al., 1999; Gentile et al., 2009; Grijalva et al., 2015; see also Hypothesis 2). Furthermore, women and men reported the same goals in using the photograph—above all, make a competent impression, provide a first impression of themselves, appear likable and/or attract attention. Hence, all variables under investigation did not explain the observed face-ism effect. Only appearance self-esteem was related to facial prominence in both women and men, but was no longer significant when analyzed separately for each gender. In any case, people with higher appearance self-esteem seemed to have less reservations about presenting their faces prominently, and used this opportunity to highlight (supposedly) desirable aspects of themselves.

However, in the present sample women tended to create a likable impression with their photograph more often than men, and men tended to use their size and strength to influence people (Intimidation) more than women. This is in line with women's communal and men's agentic gender identities. These tendencies are in accord with recent research showing that women are more likely to signal emotions (e.g., eye contact, smile intensity) on social and professional networking sites, whereas men are more likely to signal status in their portraits; e.g., more objects and formal attire; on Facebook: Tifferet and Vilnai-Yavetz, 2014; on LinkedIn: Tifferet and Vilnai-Yavetz, 2018). As higher facial prominence is associated with higher ascriptions of intelligence, competence, and dominance (e.g., Archer et al., 1983; Schwarz and Kurz, 1989; Zuckerman and Kieffer, 1994; Loughnan et al., 2010), women may have preferred to show less of their faces in order to be perceived as more likable. While this behavior may help to maintain social relationships, it may also result in being perceived as less intelligent, competent, and dominant. Future research needs to determine whether these differences in self-presentational behavior really give men an advantage over women or not and if so, in which ways. In any case, men's higher facial prominence may reinforce gender-stereotypical conceptions of men as more competent, assertive, and intelligent than women (e.g., Prentice and Carranza, 2002). The prediction of face-ism by appearance self-esteem as well as the observed tendencies among women to appear likable and among men to intimidate may be used as starting points for future research.

This research has certain limitations which also need to be mentioned: In taking the pictures, the photographers produced a minor difference in the opposite direction of face-ism, maybe because they were aware of the hypothesis and tried to avoid face-ism. Therefore, female participants had a slightly higher facial prominence than male participants in the original photographs from which they created their profile pictures. Future research should eliminate this problem by checking the face-ism indices of the original photographs before asking participants to crop them.

Furthermore, the present study was conducted by male experimenters only. It is possible that, being in male company, male participants felt more confident and were more willing to show their faces in detail. Although all instructions were provided online and little interaction took place between participants and experimenters during recruitment and photographing, it is advisable to engage both female and male experimenters in future research.

In general, people evaluate more distant pictures (half-figure, whole-figure) of themselves as more attractive than portraits (Costa and Bitti, 2000), which may result in a preference to avoid close-ups of their faces. The scenario of the present study, however, might have skewed the results in favor of higher facial prominence than would have been the case in real-life settings. In the laboratory setting, participants were asked to crop their photograph to prepare a profile picture for a professional network and to present themselves in the best possible way. Although this setting was developed to be as naturalistic as possible and comparable to the procedure at a professional photo studio, people are usually not instructed by others to prepare such pictures in real life, unless they discuss the picture detail with the photographer or other people. Therefore, future research should also investigate manifestations of face-ism in real life, for instance, by asking women and men how they select and design self-portraits for use in social media (such as profile pictures in professional networks) or for use in resumes, especially with respect to the face-to-body ratio of their portraits.

Moreover, the present research is limited to a student sample, studies with non-student populations would be necessary to generalize the present findings. This is of particular importance, as past research has shown that age and (occupational) status may moderate face-ism effects on professional networking websites (Szillis and Stahlberg, 2007; Read et al., 2017).

The observed face-ism effect in the present laboratory study is in line with face-ism effects consistently found on professional networking platforms in naturalistic studies (i.e., official websites of politicians and professors; Konrath and Schwarz, 2007; Szillis and Stahlberg, 2007; Konrath et al., 2012). Other research has also documented face-ism effects on social networking platforms (such as Facebook, MySpace, VKontakte; Reichart Smith and Cooley, 2012; Cooley and Reichart Smith, 2013). But the opposite effect has also been observed in social networks: Women displayed more facial prominence than men, in that they preferred to add portrait photos to their profiles, while men chose full-body shots (on StudiVZ, a German equivalent of Facebook; Haferkamp et al., 2012). Similarly, younger women (18 to 24 years) presented themselves with higher facial prominence than younger men on online dating sites, while no gender difference occurred in the middle-aged group (25 to 41 years) and face-ism occurred in the older-aged group (men over 41 years presented themselves with more facial prominence in their dating profiles than women over 41; Prieler and Kohlbacher, 2017). Recent research shows that people actually choose different images for dating, Facebook, and professional contexts suggesting that they aim for different impressions in the different contexts (White et al., 2017). Obviously, face-ism varies with context and with people's particular goals of self-presentation, therefore future research needs to investigate specific explanations for face-ism in the different contexts.

To conclude, the present research provides first evidence that women and men themselves create face-ism effects in their profile pictures for a professional network in social media, over and above the influence of photographers. Since facial prominence evoked by pictures in social media can have crucial positive and negative consequences for network users, it is important to further investigate face-ism and the underlying mechanisms in women's and men's self-presentation in social media.

## Author contributions

SS developed the initial research idea and the concrete study concept was generated by both authors. SS and MK performed the data analysis and interpreted the results. SS drafted the manuscript, and MK provided critical revisions. All authors approved the final version of the manuscript for submission.

### Conflict of interest statement

The authors declare that the research was conducted in the absence of any commercial or financial relationships that could be construed as a potential conflict of interest.

## References

[B1] Abele A. E. Wojciszke B. (2014). Communal and agentic content in social cognition: a dual perspective model, in Advances in Experimental Social Psychology, Vol. 50, eds Zanna M. P. Olson J. M. (Burlington, MA: Academic Press), 195–255

[B2] Archer D. Iritani B. Kimes D. D. Barrios M. (1983). Face-ism: Five studies of sex differences in facial prominence. J. Pers. Soc. Psychol. 45, 725–735. 10.1037/0022-3514.45.4.725

[B3] Cheek N. N. (2016). Face-ism and objectification in mainstream and LGBT magazines. PLoS ONE 11:e0153592. 10.1371/journal.pone.0153592 27074012 PMC4830510

[B4] Cooley S. C. Reichart Smith L. (2013). Presenting me! an examination of self-presentation in US and Russian online social networks. Russ. J. Commun. 5, 176–190. 10.1080/19409419.2013.805671

[B5] Copeland G. A. (1989). Face-ism and primetime television. J. Broadcast. Electron. Media 33, 209–214. 10.1080/08838158909364075

[B6] Costa M. Bitti P. E. R. (2000). Face-ism effect and head canting in one's own and others' photographs. Eur. Psychol. 5, 293–301. 10.1027//1016-9040.5.4.293

[B7] Dion K. L. Dion K. K. Keelan J. P. (1990). Appearance anxiety as a dimension of social-evaluative anxiety: exploring the ugly duckling syndrome. Contemp. Soc. Psychol. 14, 220–224.

[B8] Dodd D. H. Harcar V. Foerch B. J. Anderson H. T. (1989). Face-ism and facial expressions of women in magazine photos. Psychol. Rec. 39, 325–331. 10.1007/BF03395884

[B9] Donnelly K. Twenge J. M. (2017). Masculine and feminine traits on the Bem Sex-Role Inventory, 1993–2012: a cross-temporal meta-analysis. Sex Roles 76, 556–565. 10.1007/s11199-016-0625-y

[B10] Faul F. Erdfelder E. Lang A.-G. Buchner A. (2007). G^*^Power 3: a flexible statistical power analysis program for the social, behavioral, and biomedical sciences. Behav. Res. Methods 39, 175–191. 10.3758/BF03193146 17695343

[B11] Fox J. Rooney M. C. (2015). The dark triad and trait self-objectification as predictors of men's use and self-presentation behaviors on social networking sites. Pers. Individ. Dif. 76, 161–165. 10.1016/j.paid.2014.12.017

[B12] Freeman J. B. Ambady N. (2011). A dynamic interactive theory of person construal. Psychol. Rev. 118, 247–279. 10.1037/a0022327 21355661

[B13] Gentile B. Grabe S. Dolan-Pascoe B. Twenge J. M. Wells B. E. Maitino A. (2009). Gender differences in domain-specific self-esteem: a meta-analysis. Rev. Gen. Psychol. 13, 34–45. 10.1037/a0013689

[B14] Gillespie B. L. Eisler R. M. (1992). Development of the feminine gender role stress scale: A cognitive-behavioral measure of stress, appraisal, and coping for women. Behav. Modif. 16, 426–438. 10.1177/01454455920163008 1627123

[B15] Grijalva E. Newman D. A. Tay L. Donnellan M. B. Harms P. D. Robins R. W.. (2015). Gender differences in narcissism: a meta-analytic review. Psychol. Bull. 141, 261–310. 10.1037/a0038231 25546498

[B16] Guadagno R. E. Cialdini R. B. (2007). Gender differences in impression management in organizations: a qualitative review. Sex Roles 56, 483–494. 10.1007/s11199-007-9187-3

[B17] Haferkamp N. Eimler S. C. Papadakis A.-M. Kruck J. V. (2012). Men are from mars, women are from venus? Examining gender differences in self-presentation on social networking sites. Cyberpsychol. Behav. Soc. Netw. 15, 91–98. 10.1089/cyber.2011.0151 22132897

[B18] Iijima Hall C. C. Crum M. J. (1994). Women and “body-isms” in television beer commercials. Sex Roles 31, 329–337. 10.1007/BF01544592

[B19] Konrath S. Au J. Ramsey L. R. (2012). Cultural differences in face-ism: male politicians have bigger heads in more gender-equal cultures. Psychol. Women Q. 36, 476–487. 10.1177/0361684312455317

[B20] Konrath S. H. Schwarz I. (2007). Do male politicians have big heads? Face-ism in online self-representations of politicians. Media Psychol. 10, 436–448. 10.1080/15213260701533219

[B21] Leary M. R. (1995). Self-presentation: Impression Management and Interpersonal Behavior. Madison, WI: Brown and Benchmark.

[B22] Leary M. R. Kowalski R. M. (1990). Impression management: a literature review and two-component model. Psychol. Bull. 107, 34–47. 10.1037/0033-2909.107.1.34

[B23] Lee S. J. Quigley B. M. Nesler M. S. Corbett A. B. Tedeschi J. T. (1999). Development of a self-presentation tactics scale. Pers. Individ. Dif. 26, 701–722. 10.1016/S0191-8869(98)00178-0

[B24] Loughnan S. Haslam N. Murnane T. Vaes J. Reynolds C. Suitner C. (2010). Objectification leads to depersonalization: the denial of mind and moral concern to objectified others. Eur. J. Soc. Psychol. 40, 709–717. 10.1002/ejsp.755

[B25] Matthews J. L. (2007). Hidden sexism: facial prominence and its connections to gender and occupational status in popular print media. Sex Roles 56, 515–525. 10.1007/s11199-007-9276-3

[B26] Mendelson A. L. Papacharissi Z. (2010). Look at us: collective narcissism in college student facebook photo galleries, in The Networked Self: Identity, Community and Culture on Social Network Sites, ed Papacharissi Z. (London: Routledge), 251–273.

[B27] Nigro G. N. Hill D. E. Gelbein M. E. Clark C. L. (1988). Changes in the facial prominence of women and men over the last decade. Psychol. Women Q. 12, 225–235. 10.1111/j.1471-6402.1988.tb00938.x

[B28] Prentice D. A. Carranza E. (2002). What women and men should be, shouldn't be, are allowed to be, and don't have to be: the contents of prescriptive gender stereotypes. Psychol. Women Q. 26, 269–281. 10.1111/1471-6402.t01-1-00066

[B29] Prieler M. Kohlbacher F. (2017). Face-ism from an international perspective: gendered self-presentation in online dating sites across seven countries. Sex Roles. 77, 604–614. 10.1007/s11199-017-0745-z

[B30] Raskin R. Terry H. (1988). A principal-components analysis of the narcissistic personality inventory and further evidence of its construct validity. J. Pers. Soc. Psychol. 54, 890–902. 10.1037/0022-3514.54.5.890 3379585

[B31] Read G. L. Pavelko R. L. Hwang H. (2017). Social and evolutionary explanations for Face-ism: facial prominence in female academic profile pictures. J. Commun. Res. Rep. 34, 98–105. 10.1080/08824096.2016.1236331

[B32] Reichart Smith L. Cooley S. C. (2012). International faces: an analysis of self-inflicted face-ism in online profile pictures. J. Intercult. Commun. Res. 41, 279–296. 10.1080/17475759.2012.728771

[B33] Schütz A. Marcus B. Sellin I. (2004). Die Messung von Narzissmus als Persönlichkeitskonstrukt [Measuring narcissism as a personality construct]. Diagnostica 50, 202–218. 10.1026/0012-1924.50.4.202

[B34] Schwarz N. Kurz E. (1989). What's in a picture? The impact of face-ism on trait attribution. Eur. J. Soc. Psychol. 19, 311–316. 10.1002/ejsp.2420190405

[B35] Sparks G. G. Fehlner C. L. (1986). Faces in the news: gender comparisons of magazine photographs. J. Commun. 4, 70–79. 10.1111/j.1460-2466.1986.tb01451.x

[B36] Szillis U. Stahlberg D. (2007). The face-ism effect in the internet. differences in facial prominence of women and men. Int. J. Internet Sci. 2, 3–11.

[B37] Tifferet S. Vilnai-Yavetz I. (2014). Gender differences in facebook self-presentation: an international randomized study. Comput. Hum. Behav. 35, 388–399. 10.1016/j.chb.2014.03.016

[B38] Tifferet S. Vilnai-Yavetz I. (2018). Self-presentation in LinkedIn portraits: common features, gender, and occupational differences. Comput. Hum. Behav. 80, 33–48. 10.1016/j.chb.2017.10.013

[B39] Unger R. K. Crawford M. E. (1992). Women and Gender: A Feminist Psychology. Philadelphia, PA: Temple University Press.

[B40] White D. Sutherland C. A. M. Burton A. L. (2017). Choosing face: the curse of self in profile image selection. Cogn. Res. Princ. Implic. 2:23. 10.1186/s41235-017-0058-3 28470036 PMC5391387

[B41] Zebrowitz L. A. (2006). Finally, faces find favor. Soc. Cogn. 24, 657–701. 10.1521/soco.2006.24.5.657

[B42] Zuckerman M. Kieffer S. C. (1994). Race differences in face-ism: does facial prominence imply dominance? J. Pers. Soc. Psychol. 66, 86–92.

